# Nephrotic Syndrome Associated With Heavy Metals Exposure: A Case Report and Literature Review

**DOI:** 10.7759/cureus.52029

**Published:** 2024-01-10

**Authors:** Petros Kalogeropoulos, Aggeliki Sardeli, George Liapis, Panagiotis Giannakopoulos, Sophia Lionaki

**Affiliations:** 1 Department of Nephrology, Second Department of Propaedeutic Internal Medicine, Attikon University Hospital, Medical School, National and Kapodistrian University of Athens, Athens, GRC; 2 First Department of Pathology, Medical School, National and Kapodistrian University of Athens, Athens, GRC

**Keywords:** lead, toxicity, steroid-resistant, nephrotic syndrome, environmental exposure

## Abstract

Heavy metals are found in many products used in everyday life. In addition, many workers are exposed to higher concentrations of such metals in their work environment. Many of these metals may cause toxic effects in humans and there are many reports relating them to the occurrence of kidney disorders such as nephrotic syndrome. In this study, we present a case of a 38-year-old woman with nephrotic syndrome suspected to be related to heavy metal toxicity, after ruling out all other secondary causes. At the same time, she proved refractory to multiple therapies. Furthermore, a related literature review regarding the occurrence of nephrotic syndrome in patients with heavy metal exposure is presented with emphasis on the importance of considering them as a secondary cause, especially in cases that appear resistant to treatment.

## Introduction

Heavy metals are chemical elements that have a high atomic weight and density. Their distribution in the environment is widespread, either due to natural occurrences or their utilization in various human activities. The main activities include industry, agriculture, medicine, and various technological applications. They can also be found in everyday life, present in products we use or consume regularly, such as drinking water and cosmetic products. However, this occurrence is now extremely rare, given the widespread availability of purified drinking water in both urban and rural areas [1-3]. These metals are toxic to living organisms and may have adverse effects on human health. These include cardiovascular diseases, neurological disorders, developmental disorders, diabetes mellitus, immunologic and allergic disorders, kidney disorders, and various types of cancer. Exposure can occur by various routes such as inhalation, ingestion, and skin contact. The severity of the effects depends on the type of metal, the chemical form in which it is found, the time of exposure, and the amount that the human body encounters. For some elements, harm is caused only if the concentration in the human body is higher than needed (i.e., manganese, zinc, and copper) [2,4,5]. For others, such as mercury and lead, even small amounts can cause toxicity [1-3]. Through the years, nephrotic syndrome has been related to exposure to certain heavy metals [5]. Previous case reports and literature have shed light on this correlation and thus should be taken into consideration in cases that present with nephrotic syndrome of unknown origin [5]. The heavy metal that is predominantly studied and associated with nephrotic syndrome, particularly in the context of membranous nephropathy (MN) and minimal change disease (MCD), is mercury [5]. The most frequent exposure is due to medication, lightening creams, and hair dyes [6-9]. Here, we present a case of a female who came to our attention as a referral seeking a second opinion for severe, treatment-resistant nephrotic syndrome, with suspicion of heavy metal intoxication. A thorough review of the literature on the reports of heavy metal exposure and nephrotic syndrome is also presented.

## Case presentation

A 38-year-old white female was referred to our department for a second opinion and evaluation due to long-standing and treatment-resistant nephrotic syndrome. At the time of referral, she presented with nephrotic syndrome and chronically impaired renal function, indicated by a serum creatinine level of 1.5 mg/dL and an estimated glomerular filtration rate (eGFR using CKD-EPI) of 39 mL/minute/1.73 m². She had undergone two kidney biopsies and had received multiple courses of immunosuppressive therapies, including glucocorticoids alone on two occasions, as well as a combination of cyclosporine with glucocorticoids and rituximab. Her initial presentation occurred at the age of 33 with nephrotic syndrome and normal renal function. At that point, a comprehensive workup for secondary causes of nephrotic syndrome was conducted, which included tests for quantitative immunoglobulins, serum and urine protein electrophoresis, hepatitis B, hepatitis C, and human immunodeficiency virus, antiphospholipid antibody, antinuclear antibody, antineutrophil cytoplasmic antibody, and rheumatoid factor. All results were negative. According to the findings from the first kidney biopsy, the glomeruli exhibited no significant abnormalities. The interstitium appeared normal, showing no signs of interstitial inflammation, fibrosis, or tubular atrophy. Additionally, immunofluorescence examination yielded negative results for all markers, including immunoglobulins G (IgG), IgA, and IgM; complement components C3, C1q, and k; and l light chains (DAKO, Fluorescein Isothiocyanate [FITC], and Polyclonal Rabbit 1/50 dilution). Pathology examination findings, in association with the presence of nephrotic syndrome, were suggestive of podocytopathy, MCD, or *unsampled* focal segmental glomerulosclerosis (FSGS) (due to a relatively small number of glomeruli in the tissue sample - 14 glomeruli). In the absence of evidence of a secondary cause, a provisional diagnosis of idiopathic podocytopathy was made and high-dose prednisone was administered combined with angiotensin-converting enzyme inhibitors, diuretics, statins, and prophylactic anticoagulation. However, she did not respond, and treatment was changed to cyclosporine (3 mg/kg/day) combined with low-dose methylprednisolone. The patient, employed in an office job, underwent evaluation by the company's occupational physician. The occupational physician recommended a workup for heavy metal exposure and/or intoxication. This involved the collection of blood and hair samples, following specific preparation and instructions. Laboratory testing included an extensive list of heavy metals, including the most commonly toxic to humans such as chromium, arsenic, lead, cadmium, and mercury. At that point, she was found to have high levels of lead in the hair sample (55 mcg/g, normal range <30 mcg/g). However, blood lead levels were within the normal range (1.6 mcg/dL, with an elevated reference value >3.5 mcg/dL). Notably, there were elevated zinc protoporphyrin (ZPP) levels in the blood (67.1 μmol/mol, normal value <40 μmol/mol). Furthermore, measurements for mercury in both blood and urine yielded negative results. Αll of the aforementioned laboratory findings could document a previous exposure to lead in the previous months. The patient received no specific treatment or chelation therapy but was advised by a toxicologist to change the place of accommodation, which she did. Notably, she was working from home.

**Figure 1:**
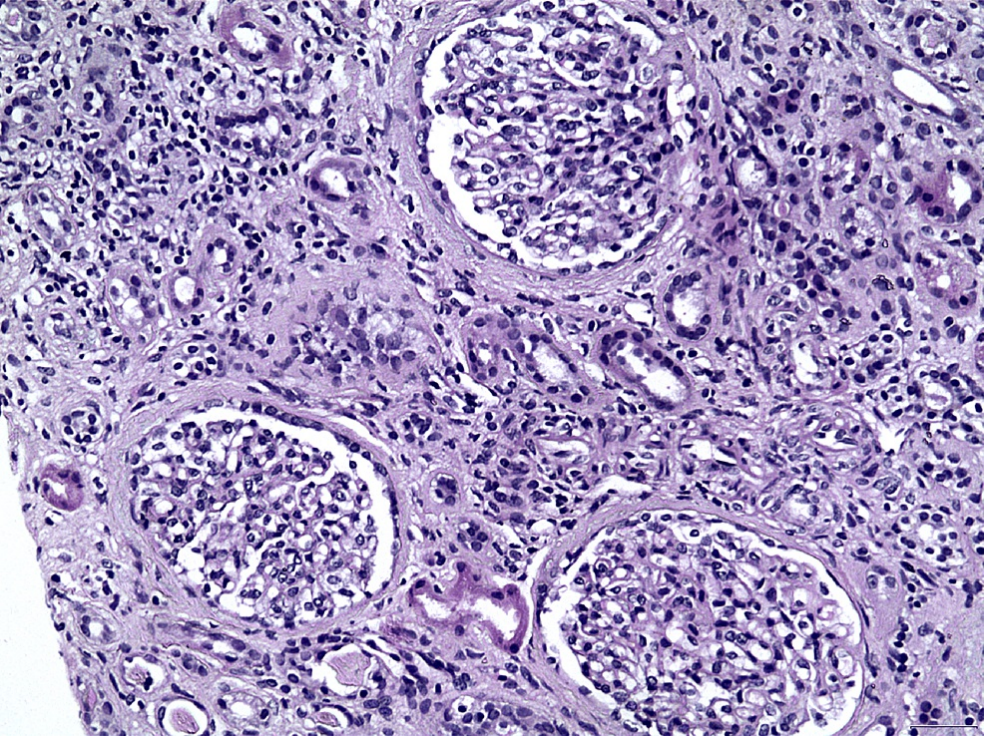
Active tubulointerstitial inflammation with areas of tubulitis, as well as moderate interstitial fibrosis and tubular atrophy (H&E x100). H&E, hematoxylin and eosin

**Figure 2:**
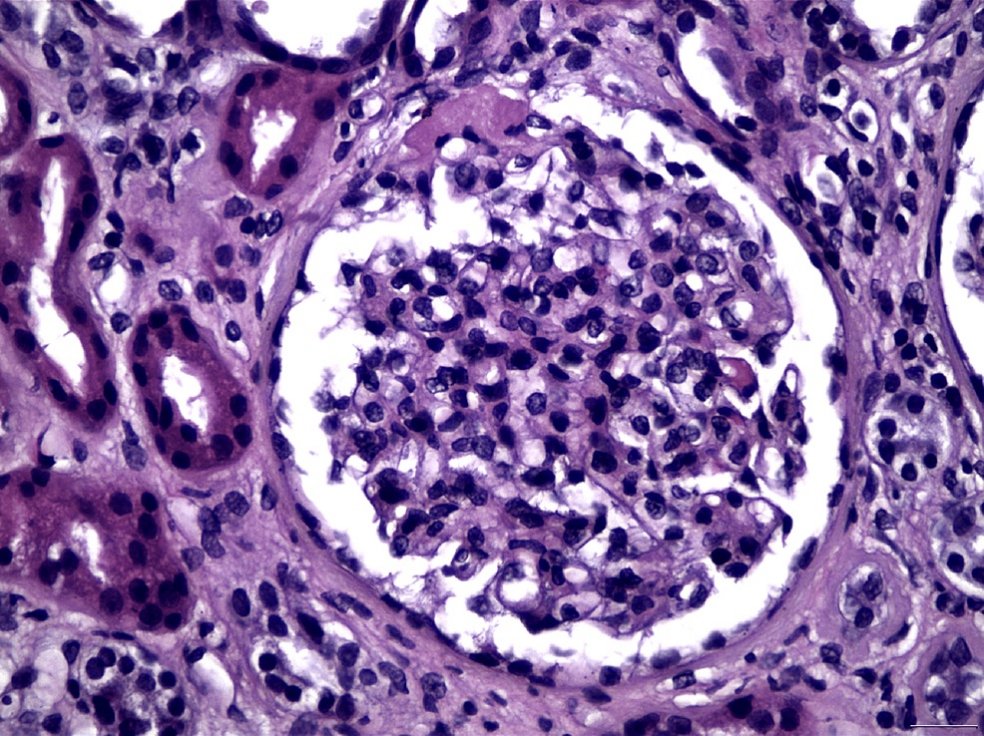
Arteriolohyalinosis in the vascular pole of a glomerulus (H&E x400). H&E, hematoxylin and eosin

**Figure 3:**
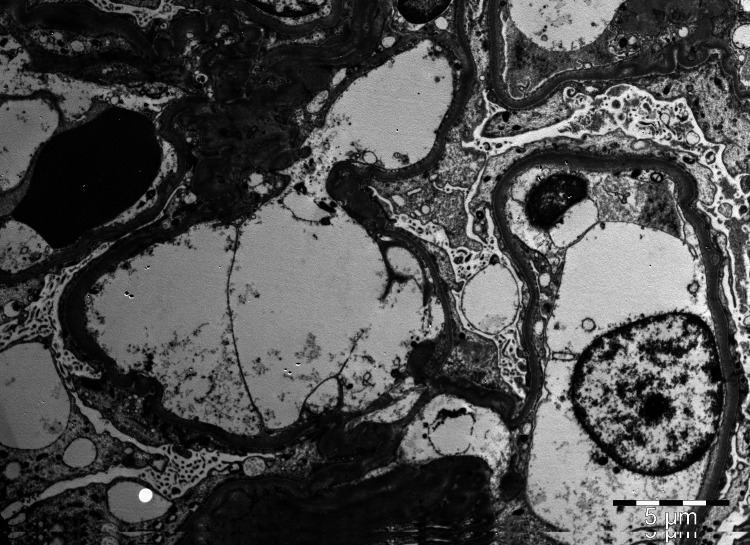
Diffuse foot processes effacement with microvilli transformation; no electron dense deposits were found (EM, uranyl acetate x3500). EM, electron microscopy

Simultaneously, despite ongoing immunosuppressive therapy, the nephrotic syndrome persisted. Consequently, she underwent a second kidney biopsy one year after the initial procedure. As per the findings from the second biopsy, certain glomeruli exhibited mild-to-moderate ischemic changes, characterized by thickening of Bowman's capsule and mild wrinkling of the glomerular basement membranes. There was also active tubulointerstitial inflammation, consisting of lymphocytes, monocytes, and a few plasma cells, associated with focal tubulitis. In addition, moderate interstitial fibrosis and tubular atrophy were demonstrated (Figure 1). Arterioles showed focally moderate artheriolohyalinosis (Figure 2). Immunofluorescence examination was negative again, for all the same markers used in the first biopsy. Examination through electron microscopy revealed diffuse foot process effacement with microvilli transformation of foot processes, along with podocyte activation (Figure 3). No electron-dense deposits were found. As a result, the nephrotic syndrome was attributed to podocytopathy again. The presence of focal arteriolohyalinosis was suspicious for cyclosporine toxicity. Active tubulointerstitial nephritis was recorded in the pathology report and thorough clinical examination and correlation were advised. Rituximab combined with oral prednisolone (0.5 mg/kg/day) was administered to her as a second-line therapy. Unfortunately, the patient never achieved remission of nephrotic syndrome. Renal function gradually declined, with an eGFR using the Chronic Kidney Disease Epidemiology Collaboration (CKD-EPI) formula of 36 mL/minute/1.73 m², while proteinuria persisted in the full nephrotic range (mean value of 16.5 g/day). Upon referral to our department, she underwent a reevaluation for secondary causes of nephrotic syndrome, with a focus on hematological malignancies, cancer, infections, and autoimmune disorders. No findings were identified. As the aforementioned secondary causes were ruled out, genetic screening was performed using the Whole Exome Sequencing (WES) technique. The results were negative, and no correlation was made with any of the known genes that cause FSGS, including *NPHS1*, *NPHS2*, *ACTN4*, *INF2*, *COL4A3*, *COL4A4*, *COL4A5*, *CD2AP*, *LMX1B*, and *APOL1* genes. The patient was re-tested for heavy metal exposure. Whole blood mercury concentrations, 24-hour urine for mercury measurement, blood lead levels, and blood ZPP were obtained from the patient. Remarkably, a repeat of investigations regarding heavy metals carried out in the same laboratory was negative. Considering the history of resistance to multiple immunosuppressive therapies, the advanced stage of CKD (histopathologically confirmed), and the presence of significant side effects such as posterior subscapular cataracts, we advised the patient against receiving further immunosuppression.

## Discussion

Glomerulopathies, such as MN and MCD, have been associated with exposure to heavy metals. A few cases have been reported over the years, with an established correlation between them. The metal most frequently reported to be associated with such clinical conditions is mercury. The mechanism by which mercury causes glomerulopathies is not fully understood. It has been suggested that in patients exhibiting such glomerulopathies, mercury induces immunomodulatory activity, activating an immune response to the heavy metal [10,11]. Several studies showed that low concentrations of mercury induced activation of both cellular and humoral immunity in rats [12-14]. Diagnosis of elemental mercury toxicity should be suspected among individuals who present with consistent clinical manifestations and who have a history of exposure risk via occupation or have days to weeks of exposure to elemental mercury via a broken mercury-containing device or other mercury spill [15-18]. Diagnosis is confirmed by measuring whole blood mercury levels and 24-hour urine levels. Acute mercury poisoning can mainly affect the respiratory system, the central nervous system, the digestive system, and the skin while also causing acute tubular necrosis [19,20]. Patients with more severe symptoms and elevated mercury levels are candidates for chelator treatment beyond supportive care [21,22]. Patients who have developed renal symptoms such as proteinuria do not need any special treatment as long as they are not symptomatic from other systems. The most important action is to eliminate or minimize exposure to mercury as nephrotic syndrome due to mercury is usually reversible, although it may take several months [11,23]. In a case series with 11 patients with MN, authors concluded that there was significant mercury exposure before MN diagnosis [24]. Exposure to heavy metals was related to the use of mercury-containing pills, hair-dyeing agents, skin-lightening cream, or mercury vapor. All patients were admitted with proteinuria and normal renal function, whereas three of them had nephrotic syndrome. Yet, MN associated with mercury exposure had a different pathogenesis [24]. There are also other reports linking the occurrence of nephrotic syndrome to cosmetic products containing mercury [25-29]. In a case report, a male with a history of mercury exposure, who presented with nephrotic syndrome and microscopic hematuria, was found to have an MN in the renal biopsy. All other secondary causes for MN were excluded, and the patient responded to treatment with both chelation and immunosuppression. The patient had been working as an operator in a company of copper enamel for two years [30]. A report described eight cases with biopsy-proven MN and exposure to mercury, where mercury intake was shown to be related to indigenous medications, administered mostly for asthma, upper respiratory tract infections, and allergic symptoms [6]. All eight patients developed proteinuria and edema. Another similar report from India described five cases of ΜΝ due to chronic mercury poisoning from traditional Indian medicines [31]. Interestingly, a 14-year-old male adolescent was diagnosed with MN attributed to mercury intoxication. Though MN is not common in children, this adolescent appeared with stage III CKD, nephrotic syndrome, and a kidney biopsy confirming MN. The patient had recently moved to a trailer home where mercury from spilled bottles on countertops was suspected to be the cause. Mercury levels in both blood and urine were measured, revealing abnormal levels, while other potential causes for primary or secondary MN were not identified [32]. Another study, which analyzed the characteristics of 35 cases of mercury-induced glomerulonephritis in a Chinese center, reported that mercury exposure was associated with skin-lightening creams in 20 patients, mercury-containing pills in nine patients, hair-dyeing agents in four patients, and unidentified reasons in two. All patients presented with proteinuria and normal renal function. Of these, 22 patients presented with nephrotic syndrome, 21 had MCD, 13 had MN, and 1 had FSGS, all associated with exposure to mercury [33]. A 39-year-old male with MN was associated with exposure to both mercury and arsenic. This patient presented with bilateral lower-extremity edema and abdominal distention while regularly taking multiple oral herbal supplements daily, which contained mercury and arsenic [34]. Interestingly, a female patient diagnosed with Guillain-Barre syndrome later developed nephrotic syndrome due to MN. She had daily exposure to skin-lightening creams and hair dyes containing mercury, probably causing neuropathy and glomerulonephritis [35]. A review of 30 subjects with nephrotic syndrome and a history of mercury exposure found that renal histopathology revealed MCD in the majority and MN in the remaining cases. Spontaneous remission was rare, with suggested treatments including chelation therapy among others [8]. In a case report by Gao et al., a 65-year-old Asian woman developed nephrotic syndrome associated with the use of mercury-containing skin-lightening cream. Renal biopsy revealed MCD with IgA deposition. After treatment with sodium dimercaptopropane sulfonate and discontinuation of the skin-lightening cream, the patient demonstrated complete remission of nephrotic syndrome. She did not receive any glucocorticoid treatment [36]. Another case involves a 42-year-old man who developed nephrotic syndrome eight months after attempting suicide with elemental mercury. The histopathological report of the biopsy revealed MCD. The patient was treated with 2,3-dimercaptopropane-1-sulfonate (DMPS), for mercury detoxification, and steroids, and one year later he had complete remission of the nephrotic syndrome and normal kidney function [37]. In a retrospective analysis of patients diagnosed with mercury poisoning, 41 patients presented with nephrotic syndrome. Thirty-five patients underwent renal biopsy, of which 18 had ΜΝ and 13 had MCD. All patients underwent chelation therapy with or without immunosuppression. Of 41 patients, 40 had complete remission of nephrotic syndrome regardless of treatment [38]. A 25-year-old man presented with nephrotic syndrome after occupational exposure to mercury vapor. The kidney biopsy revealed MCD, and the patient was administered chelation therapy with DMPS along with steroids, leading to the complete resolution of his nephrotic syndrome [39]. Finally, another report mentioned inhalation and vapor mercury poisoning in a family of four members. Three of them developed, among other symptoms, nephrotic syndrome [40].

Lead is another agent that can affect the kidneys. There are various sources of lead exposure, including occupational exposure in adults, as well as inhalation or ingestion of environmental lead in both adults and children. However, exposure at a high level is very rare in developed countries. Acute and high levels of lead exposure injure the proximal tubules, and patients present with signs of Fanconi syndrome, including glucosuria, aminoaciduria, and phosphaturia [41,42]. With chronic high-level exposure, patients typically present with impaired kidney function, relatively normal urine sediment, little or no proteinuria, and hyperuricemia [43,44]. Kidney biopsy reveals the typical findings of chronic interstitial nephritis, which includes interstitial fibrosis and tubular atrophy, findings consistent with the interstitial fibrosis seen in the patient's second biopsy [41,45,46]. Lead nephropathy should be considered in the differential diagnosis for patients who present with the triad of CKD, hypertension, and gout. Additionally, it should be taken into account for patients with CKD exhibiting symptoms and signs of poisoning in other organs, including gastrointestinal, musculoskeletal, neuropsychiatric, and anemia [47]. Blood lead levels (BLLs) should be obtained, as it is the key test to determine the extent of lead absorption in a patient. It reflects recent exposure to exogenous lead sources and the release of endogenous lead from bone and soft tissue stores. This test is crucial in guiding treatment decisions [47]. The measurement of lead levels in hair or urine is not as accurate as BLLs and does not correlate as well with adverse effects [47]. ZPP is a useful biomarker of lead exposure in individuals with low BLLs, concentrations that are not high enough to account for the symptoms, especially when there is suspicion that BLL may have been higher during the preceding three or four months. It is important to note that ZPP levels can be elevated in the presence of iron deficiency anemia, jaundice, and sickle cell anemia [47,48].

However, all of the aforementioned conditions were excluded in our patient's case, thus making the increased value of ZPP suggestive of the diagnosis of previous lead exposure. Also, exposure to lead even at lower concentrations can cause lead-related nephrotoxicity, leading to a gradual decrease in GFR and an increase in albuminuria [49,50]. The mechanism by which low levels of lead exposure accelerate the progression of CKD is unknown, but this exposure can lead to glomerular hyperfiltration, as has been noted in animal models [51]. While upper renal manifestations are common, there are few reports linking lead to nephrotic syndrome. In the case report by Tang et al., a 42-year-old man presented with nephrotic syndrome and worsening renal function requiring renal replacement therapy. The patient had undergone a renal biopsy 20 years ago due to nephrotic syndrome, which had revealed MCD. He underwent a second renal biopsy, which revealed FSGS. The patient worked as an electrical welder and had elevated blood levels of cadmium and lead. He resigned from his job due to possible toxicity, and after one year of follow-up, the patient recovered partial kidney function with negative screening for the aforementioned metals in blood and urine [52]. Furthermore, an observational epidemiologic study compared the occupations and toxic exposures of 100 patients with histologically confirmed MN with those of the general population. The study found that patients more often worked in the construction sector and were exposed to toxic substances such as asbestos, lead, and organic solvents [53].

However, other agents implicated in kidney disorders include thallium, cadmium, and chromium. Six pediatric patients presented with impaired renal function and proteinuria after exposure to thallium [54]. In addition, a study involving 5,426 subjects over more than 20 years found that elevated levels of blood and urinary cadmium were associated with increased albuminuria and CKD [55]. At the same time, there is evidence that arsenic, which is frequently found in water and soil, may correlate with CKD, but more studies are necessary to prove this [56]. Interestingly, chromium, lead, and cadmium were investigated for their association with renal function, measured using eGFR [57]. Among 360 adults from Taiwan who were studied, a decline in eGFR was shown when chromium or lead level was doubled. For those with cadmium exposure, there was a higher decline in eGFR [57]. All reports of patients with nephrotic syndrome and heavy metal exposure are summarized in Table 1.

**Table 1:** Reports of patients with nephrotic syndrome and heavy metal exposure. MN, membranous nephropathy; FSGS, focal segmental glomerulosclerosis; IgAN, IgA nephropathy; MCD, minimal change disease; MesPGN, mesangial proliferative glomerulonephritis; NA, not available

First author (year)	Implicated heavy metal	Number of patients	Histopathological diagnosis	Clinical presentation
Pelclová et al. (2002) [28]	Mercury	1	NA	Nephrotic syndrome
Soo et al. (2003) [26]	Mercury	1	MN	Nephrotic syndrome
Tang et al. (2006) [27]	Mercury	1	MCD	Nephrotic syndrome
Campbell et al. (2009) [39]	Mercury	1	MCD	Nephrotic syndrome
Li et al. (2010) [24]	Mercury	11	MN	Proteinuria and nephrotic syndrome (3)
Oz et al. (2012) [40]	Mercury	3	NA	Nephrotic syndrome
Tang et al. (2013) [29]	Mercury	4	MCD	Nephrotic syndrome
Miller et al. (2013) [9]	Mercury	1	FSGS	Nephrotic syndrome
Voitzuk et al. (2014) [30]	Mercury	1	MN	Nephrotic syndrome
Wągrowska-Danilewicz et al. (2014) [37]	Mercury	1	MCD	Nephrotic syndrome
Zhang et al. (2014) [25]	Mercury	1	MCD	Nephrotic syndrome
Duan et al. (2015) [54]	Thallium	6	NA	Impaired kidney function and proteinuria
Niu et al. (2017) [7]	Mercury	1	IgAN MCD	Nephrotic syndrome
Onwuzuligbo et al. (2018) [32]	Mercury	1	MN	Impaired kidney function and nephrotic syndrome
Doshi et al. (2018) [31]	Mercury	5	MN	Nephrotic syndrome
Qin et al. (2019) [33]	Mercury	35	MCD (22), MN (13), FSGS (1)	Proteinuria and nephrotic syndrome (22)
Chan et al. (2020) [8]	Mercury	30	MCD (20), MN (10)	Nephrotic syndrome
Tanner et al. (2020) [34]	Mercury and arsenic	1	MN	Nephrotic syndrome
Kumar et al. (2020) [6]	Mercury	8	MN	Proteinuria
Yawei et al. (2021) [35]	Mercury	1	MN	Nephrotic syndrome
Tang et al. (2022) [52]	Lead and cadmium	1	MCD, FSGS	Nephrotic syndrome
Gao et al. (2022) [36]	Mercury	1	MCD	Nephrotic syndrome
Gao et al. (2022) [38]	Mercury	41	MN (18), MCD (13), MesPGN (3), IgAN (1), no biopsy (6)	Nephrotic syndrome
Cremoni et al. (2022) [53]	Asbestos, lead, and organic solvent	100	MN	Nephrotic syndrome

## Conclusions

Heavy metal exposure should always be considered in the differential diagnosis, particularly in patients refractory to immunosuppression, when the most common secondary causes have been ruled out, and genetic testing yields negative results. In our case, we speculate that the podocytopathy is related to lead toxicity, with the agent being removed from the patient's environment but causing chronic and irreversible damage to the kidney. Heavy metals are present in various products used in everyday life and are commonly encountered in the working environments of individuals, particularly those in the construction sector. In many instances, recognizing heavy metal exposure presents a challenge for clinicians. There are correlations between many heavy metals, primarily mercury, and the occurrence of nephrotic syndrome. It is very important to consider them as a secondary cause of nephrotic syndrome because toxic agent removal from the patient's environment may be vital for long-term kidney prognosis. Additional studies and research are needed to investigate the role of heavy metals in everyday products, water, and the environment to enlighten and understand their association with kidney disease.

## References

[1] Viau C, Bernard A, Lauwerys RR, Tulkens P, Laurent G, Maldague P (1983). Gentamicin nephrotoxicity in cadmium, lead and mercury pretreated rats. Toxicology.

[2] Moel DI, Kumar K (1982). Reversible nephrotoxic reactions to a combined 2,3-dimercapto-1-propanol and calcium disodium ethylenediaminetetraacetic acid regimen in asymptomatic children with elevated blood lead levels. Pediatrics.

[3] Vyskocil A, Fiala Z, Salandová J, Popler A, Ettlerová E, Emminger S (1991). The urinary excretion of specific proteins in workers exposed to lead. Arch Toxicol Suppl.

[4] Chia KS, Mutti A, Alinovi R, Jeyaratnam J, Tan C, Ong CN, Lee E (1994). Urinary excretion of tubular brush-border antigens among lead exposed workers. Ann Acad Med Singap.

[5] Buchet JP, Lauwerys R, Roels H, Bernard A (1980). Relationship between exposure to heavy metals and prevalence of renal dysfunction. Arch Toxicol Suppl.

[6] Kumar MN, Priyamvada PS, Chellappan A (2020). Membranous nephropathy associated with indigenous Indian medications containing heavy metals. Kidney Int Rep.

[7] Niu HX, Li SH, Li HY, Chen YH, Liu WW, Li PL, Long HB (2017). Clinicopathological features, diagnosis, and treatment of IgA nephropathy with minimal change disease related to exposure to mercury-containing cosmetics: a case report. Clin Nephrol.

[8] Chan TY, Chan AP, Tang HL (2020). Nephrotic syndrome caused by exposures to skin-lightening cosmetic products containing inorganic mercury. Clin Toxicol (Phila).

[9] Miller S, Pallan S, Gangji AS, Lukic D, Clase CM (2013). Mercury-associated nephrotic syndrome: a case report and systematic review of the literature. Am J Kidney Dis.

[10] Moszczyński P (1997). Mercury compounds and the immune system: a review. Int J Occup Med Environ Health.

[11] Kazantzis G, Schiller KF, Asscher AW, Drew RG (1962). Albuminuria and the nephrotic syndrome following exposure to mercury and its compounds. Q J Med.

[12] Vas J, Monestier M (2008). Immunology of mercury. Ann N Y Acad Sci.

[13] Hu H, Möller G, Abedi-Valugerdi M (1999). Mechanism of mercury-induced autoimmunity: both T helper 1- and T helper 2-type responses are involved. Immunology.

[14] Rowley B, Monestier M (2005). Mechanisms of heavy metal-induced autoimmunity. Mol Immunol.

[15] Esdaile LJ, Chalker JM (2018). The mercury problem in artisanal and small-scale gold mining. Chemistry.

[16] Koirala S, Leinenkugel K (2015). Notes from the field: acute mercury poisoning after home gold and silver smelting-Iowa, 2014. MMWR Morb Mortal Wkly Rep.

[17] Tewell M, Spoto S, Wiese M, Aleguas A, Peredy T (2017). Mercury poisoning at a home day care center — Hillsborough County, Florida, 2015. MMWR Morb Mortal Wkly Rep.

[18] Lai O, Parsi KK, Wu D (2016). Mercury toxicity presenting as acrodynia and a papulovesicular eruption in a 5-year-old girl. Dermatol Online J.

[19] Asano S, Eto K, Kurisaki E (2000). Review article: acute inorganic mercury vapor inhalation poisoning. Pathol Int.

[20] Kanluen S, Gottlieb CA (1991). A clinical pathologic study of four adult cases of acute mercury inhalation toxicity. Arch Pathol Lab Med.

[21] Zhang J (1984). Clinical observations in ethyl mercury chloride poisoning. Am J Ind Med.

[22] Böse-O’Reilly S, Drasch G, Beinhoff C, Maydl S, Vosko MR, Roider G, Dzaja D (2003). The Mt. Diwata study on the Philippines 2000-treatment of mercury intoxicated inhabitants of a gold mining area with DMPS (2,3-dimercapto-1-propane-sulfonic acid, Dimaval). Sci Total Environ.

[23] Friberg L, Hammarstrom S, Nystrom A (1953). Kidney injury after exposure to inorganic mercury. AMA Arch Ind Hyg Occup Med.

[24] Li SJ, Zhang SH, Chen HP, Zeng CH, Zheng CX, Li LS, Liu ZH (2010). Mercury-induced membranous nephropathy: clinical and pathological features. Clin J Am Soc Nephrol.

[25] Zhang L, Liu F, Peng Y, Sun L, Chen C (2014). Nephrotic syndrome of minimal change disease following exposure to mercury-containing skin-lightening cream. Ann Saudi Med.

[26] Soo YO, Chow KM, Lam CW, Lai FM, Szeto CC, Chan MH, Li PK (2003). A whitened face woman with nephrotic syndrome. Am J Kidney Dis.

[27] Tang HL, Chu KH, Mak YF (2006). Minimal change disease following exposure to mercury-containing skin lightening cream. Hong Kong Med J.

[28] Pelclová D, Lukás E, Urban P (2002). Mercury intoxication from skin ointment containing mercuric ammonium chloride. Int Arch Occup Environ Health.

[29] Tang H-L, Mak Y-F, Chu K-H, Lee W, Fung SKS, Chan TY-K, Tong K-L (2013). Minimal change disease caused by exposure to mercury-containing skin lightening cream: a report of 4 cases. Clin Nephrol.

[30] Voitzuk A, Greco V, Caputo D, Alvarez E (2014). [Toxic nephropathy secondary to occupational exposure to metallic mercury]. Medicina (B Aires).

[31] Doshi M, Annigeri RA, Kowdle PC, Subba Rao B, Varman M (2019). Membranous nephropathy due to chronic mercury poisoning from traditional Indian medicines: report of five cases. Clin Kidney J.

[32] Onwuzuligbo O, Hendricks AR, Hassler J, Domanski K, Goto C, Wolf MT (2018). Mercury intoxication as a rare cause of membranous nephropathy in a child. Am J Kidney Dis.

[33] Qin AB, Su T, Wang SX, Zhang F, Zhou FD, Zhao MH (2019). Mercury-associated glomerulonephritis: a retrospective study of 35 cases in a single Chinese center. BMC Nephrol.

[34] Tanner S, Sharma V, Jebakumar D, Narayanan M, Rao A (2020). Mercury in natural health products as a cause of membranous nephropathy. Proc (Bayl Univ Med Cent).

[35] Yawei C, Jing S, Wenju S, Yupeng L, Ping Z, Liping H (2021). Mercury as a cause of membranous nephropathy and Guillain-Barre syndrome: case report and literature review. J Int Med Res.

[36] Gao H, Liu G, He Y, Chen J (2022). Nephrotic syndrome of minimal change disease following exposure to mercury-containing skin lightening cream: A case report and literature review. Clin Nephrol.

[37] Wągrowska-Danilewicz M, Danilewicz M, Zbrog Z (2014). Mercury-induced nephrotic syndrome: a case report and review of the literature. Pol J Pathol.

[38] Gao Z, Wu N, Du X, Li H, Mei X, Song Y (2022). Toxic nephropathy secondary to chronic mercury poisoning: clinical characteristics and outcomes. Kidney Int Rep.

[39] Campbell G, Leitch D, Lewington A, Dargan PI, Baker RJ (2009). Minimal-change nephrotic syndrome due to occupational mercury vapor inhalation. Clin Nephrol.

[40] Oz SG, Tozlu M, Yalcin SS, Sozen T, Guven GS (2012). Mercury vapor inhalation and poisoning of a family. Inhal Toxicol.

[41] Goyer RA, Rhyne BC (1973). Pathological effects of lead. Int Rev Exp Pathol.

[42] Loghman-Adham M (1998). Aminoaciduria and glycosuria following severe childhood lead poisoning. Pediatr Nephrol.

[43] Bennett WM (1985). Lead nephropathy. Kidney Int.

[44] Wedeen RP, Malik DK, Batuman V (1979). Detection and treatment of occupational lead nephropathy. Arch Intern Med.

[45] Inglis JA, Henderson DA, Emmerson BT (1978). The pathology and pathogenesis of chronic lead nephropathy occurring in Queensland. J Pathol.

[46] Wedeen RP, Maesaka JK, Weiner B, Lipat GA, Lyons MM, Vitale LF, Joselow MM (1975). Occupational lead nephropathy. Am J Med.

[47] Kosnett MJ, Wedeen RP, Rothenberg SJ (2007). Recommendations for medical management of adult lead exposure. Environ Health Perspect.

[48] Parsons PJ, Reilly AA, Hussain A (1991). Observational study of erythrocyte protoporphyrin screening test for detecting low lead exposure in children: impact of lowering the blood lead action threshold. Clin Chem.

[49] Ekong EB, Jaar BG, Weaver VM (2006). Lead-related nephrotoxicity: a review of the epidemiologic evidence. Kidney Int.

[50] Wrońska-Nofer T, Pisarska A, Trzcinka-Ochocka M (2015). Scintigraphic assessment of renal function in steel plant workers occupationally exposed to lead. J Occup Health.

[51] Khalil-Manesh F, Gonick HC, Cohen AH, Alinovi R, Bergamaschi E, Mutti A, Rosen VJ (1992). Experimental model of lead nephropathy. I. Continuous high-dose lead administration. Kidney Int.

[52] Tang L, Cai Z, Wang SX, Zhao WJ (2022). Transition from minimal change disease to focal segmental glomerulosclerosis related to occupational exposure: A case report. World J Clin Cases.

[53] Cremoni M, Agbekodo S, Teisseyre M (2022). Toxic occupational exposures and membranous nephropathy. Clin J Am Soc Nephrol.

[54] Duan W, Wang Y, Li Z (2020). Thallium exposure at low concentration leads to early damage on multiple organs in children: a case study followed-up for four years. Environ Pollut.

[55] Ferraro PM, Costanzi S, Naticchia A, Sturniolo A, Gambaro G (2010). Low level exposure to cadmium increases the risk of chronic kidney disease: analysis of the NHANES 1999-2006. BMC Public Health.

[56] Robles-Osorio ML, Sabath-Silva E, Sabath E (2015). Arsenic-mediated nephrotoxicity. Ren Fail.

[57] Tsai TL, Kuo CC, Pan WH, Chung YT, Chen CY, Wu TN, Wang SL (2017). The decline in kidney function with chromium exposure is exacerbated with co-exposure to lead and cadmium. Kidney Int.

